# The systemic control of cancers by the osteoblasts

**DOI:** 10.18632/oncoscience.437

**Published:** 2018-06-28

**Authors:** Claire-Sophie Devignes, Yetki Aslan, Sylvain Provot

**Affiliations:** INSERM, Hôpital Lariboisière - Centre Viggo Petersen, 75010 Paris, France

**Keywords:** osteoblast, breast cancer, metastasis, bone, HIF

The importance of the bone microenvironment in cancer biology has been particularly well illustrated in numerous studies that emphasized the deleterious roles of the osteoclasts (the bone-resorbing cells) and of the osteoblasts (the bone-making cells) in bone metastasis. Notably, genetic alterations of the osteoblasts have been also shown to be sufficient to induce *de novo* myeloid malignancies [1, 2], indicating that these cells possess a strong oncogenic power. However, the importance of the osteoblast-lineage cells in oncogenesis outside the bone microenvironment remained unknown until recently. Osteoblasts’ function is not limited to the synthesis and mineralization of the bone matrix. These cells have been shown to control multiple physiological extra-skeletal processes in various organs (brain, pancreas, testis) in mice [3], raising the possibility that osteoblasts could also influence diseases such as cancers, outside the skeleton. Moreover, clinical data showed that women with high bone mass have a higher risk of developing breast cancers and worse prognosis (see discussion in [4]), suggesting that increased bone formation (resulting from increased osteoblast number and/or function) could influence distant neoplastic processes in the breast.

In our recent study [4], we tested this possibility and we found that mouse osterix-positive osteoblast progenitor cells (OPCs) are highly hypoxic, and that activation of the hypoxia-inducible factor (HIF) signaling in these cells increases breast cancer metastasis to bone locally, but also remotely promotes primary mammary tumor growth, and metastasis to the lungs and to other organs distant from the skeleton. We observed that genetic inactivation of HIF signaling in OPCs decreased bone formation (and thus bone mass), and also the number of OPCs expressing high levels of the cytokine CXCL12, while genetic activation of HIF increased bone mass and the number of CXCL12-positive OPCs. We found that OPCs constitute a major source of CXCL12 compared to mammary tumors and hematopoietic cells. Using pharmacological and genetic approaches we demonstrated that hypoxic OPCs release in a HIF1-depended manner CXCL12 in the bloodstream, and that OPC-derived CXCL12 activates CXCR4 to directly promote breast cancer cell proliferation and metastasis, in a systemic fashion. Interestingly, our data show that increasing bone mass by inhibiting osteoclast activity does not increase breast cancer growth and metastasis, as opposed to increased bone formation that leads to a robust systemic tumorigenic effect. This suggests that only patients who have an elevated bone mass that results from increased bone formation (and not decreased bone resorption) are at risk for breast cancer. This notion is supported by a recent clinical study showing that elevated serum levels of the marker of bone formation P1NP (N-terminal propeptide of type-1 collagen) are correlated to increased risk of metastatic recurrence in the bones of breast cancer patients [5]. Hence, measurements of bone mass, and circulating levels of CXCL12 and of bone formation markers could be clinically useful as prognostic, and perhaps as predictive markers for breast cancer.

Primary breast tumors have been shown to instigate systemic signals that mobilize bone marrow-derived cells that promote tumor growth and dissemination. Our study reveals that osteoblast-lineage cells also produce systemic signals supporting distant cancer progression. This finding is important not only because of the prognostic value of the bone-derived factors that can be detected in the bloodstream, but also because it increases our awareness of the risk associated with the exposure to bone anabolic agents, and suggests that targeting the bone microenvironment could limit breast cancer growth and metastasis. What is still unclear, however, is whether additional bone-derived factors (soluble molecules or other factors such as exosomes) promote systemic breast cancer growth and metastasis in addition to CXCL12. It is also unclear whether hypoxic OPCs remotely influence other types of distant cancers, and whether OPC-derived CXCL12 could also act indirectly in breast cancer by altering immune cell differentiation or function.

Interestingly, Rossnagl and colleagues showed that fibronectin originating from type 1 collagen-expressing (differentiating) osteoblasts inhibits the immune response against melanoma and breast cancer, by fostering the production of monocytic and granulocytic myeloid-derived suppressor cells (MDSCs) in mice [6]. Moreover, Engblom, Pfirschke and colleagues recently demonstrated that in mice lung tumors remotely activate osteocalcin-positive (terminally-differentiated) osteoblasts, which in turn foster lung tumor growth by mobilizing cancer-promoting neutrophils that share phenotypic and functional profiles with granulocytic MDSCs [7]. Interestingly, increased bone mass is observed both in mice and men with lung tumors, supporting the notion that increased bone formation (upon activation of the osteoblasts) creates a systemic tumorigenic environment. While these two studies indicate that osteoblast-lineage cells control cancer progression at least in part indirectly by mobilizing tumor-promoting myeloid cells, it is possible that fibronectin in osteoblasts and lung tumors could support the pool of OPCs and/or bone-derived CXCL12 production to promote more directly cancer growth.

Together, our work and that of Inaam Nakchbandi and Mikael Pittet laboratories provide compelling evidence that osteoblast-lineage-cells that reside in bones have the capacity to promote distant cancer progression. Using different experimental approaches, these independent studies establish that the skeleton is a critical organ in the systemic control of distant systemic cancers such as melanoma, and lung and breast carcinomas. Future work will likely tell us whether additional types of cancers are similarly influenced by osteoblasts, and bring further insight into the complex crosstalk that exists between cancers and the bone microenvironment, which will translate perhaps into novel therapeutic approaches to prevent the progression of these systemic diseases.

## References

[1] Kode A (2014). Nature.

[2] Raaijmakers MH (2010). Nature.

[3] Karsenty G (2016). Cell.

[4] Devignes CS (2018). Proc Natl Acad Sci U S A.

[5] Brown J (2018). J Natl Cancer Inst.

[6] Rossnagl S (2016). PLoS Biol.

[7] Engblom C (2017). Science.

